# Case Study of Recognition Patterns in Haunted People Syndrome

**DOI:** 10.3389/fpsyg.2022.879163

**Published:** 2022-06-08

**Authors:** James Houran, Brian Laythe

**Affiliations:** ^1^Integrated Knowledge Systems, Dallas, TX, United States; ^2^Instituto Politécnico de Gestão e Tecnologia, Porto, Portugal; ^3^Institute for the Study of Religious and Anomalous Experience, Charlestown, IN, United States

**Keywords:** anomalous experiences, entity encounters, hauntings, paranormal belief, transliminality

## Abstract

Haunted People Syndrome (HP-S) denotes individuals who recurrently report various “supernatural” encounters in everyday settings ostensibly due to heightened somatic-sensory sensitivities to dis-ease states (e.g., marked but sub-clinical levels of distress), which are contextualized by paranormal beliefs and reinforced by perceptual contagion effects. This view helps to explain why these anomalous experiences often appear to be idioms of stress or trauma. We tested the validity and practical utility of the HP-S concept in an empirical study of an active and reportedly intense ghostly episode that was a clinical referral. The case centered on the life story of the primary percipient, a retired female healthcare worker. Secondary percipients included her husband and adult daughter, all of whom reported an array of benign and threatening anomalies (psychological and physical in nature) across five successive residences. Guided by prior research, we administered the family online measures of transliminality, sensory-processing sensitivity, paranormal belief, locus of control, desirability for control, and a standardized checklist of haunt-type phenomena. The primary percipient also completed a measure of adverse childhood events and supplied an event diary of her anomalous experiences. We found reasonably consistent support for HP-S from a set of quantitative observations that compared five proposed syndrome features against the family members’ psychometric profiles and the structure and contents of their anomalous experiences. Specifically, the reported anomalies both correlated with the family’s scores on transliminality and paranormal belief, as well as elicited attributions and reaction patterns aligned with threat (agency) detection. There was also some evidence of perceptual congruency among the family members’ anomalous experiences. Putative psi cannot be ruled out, but we conclude that the family’s ordeal fundamentally involved the symptoms and manifestations of thin (or “permeable”) mental boundary functioning in the face of unfavorable circumstances or overstimulating environments and subsequently acerbated by poor emotion regulation, histrionic and catastrophizing reactions, and active confirmation biases.

## Introduction

This paper examines a real-life and rather remarkable “ghost story” via a mixed methods approach that continues our series of studies about people who claim to be haunted by anomalous beings or sentient forces (Laythe et al., 2018; Drinkwater et al., 2019; Houran et al., 2019a,b; O’Keeffe et al., 2019; Ventola et al., 2019; Lange et al., 2020). Some research suggests that outwardly disparate “(entity) encounter experiences”—e.g., spirits, angels, gods, demons, poltergeists, extraterrestrials, Men in Black (MIB), and folklore–type little people—generally have similar narrative structures (Evans, 1987; Hufford, 2001; Young, 2018) and perceptual contents (Houran, 2000; Houran and Lange, 2001b; Houran et al., 2019a). However, the exact attribution or meaning of these occurrences typically reflects the percipient’s religio–cultural milieu, with many people ascribing their experiences to hauntings or poltergeists (collectively termed *ghostly episodes*) (Hill et al., 2018, 2019; Houran et al., 2019a).

To clarify, poltergeist disturbances are clusters of anomalous *subjective* (*S*) experiences (e.g., apparitions, sensed presences, hearing voices, and unusual somatic or emotional manifestations) and *objective* (*O*) events (e.g., object displacements, malfunctioning electrical or mechanical equipment, and inexplicable percussive sounds like raps or knocks), which focus on the presence of certain people (for a recent discussion, see Ventola et al., 2019). Similar *S/O* anomalies that seemingly persist at specific locations are called hauntings (Houran and Lange, 2001a). Researchers traditionally differentiate haunts and poltergeists, but the *S/O* anomalies that characterize each occurrence collectively form a probabilistic and unidimensional hierarchy (Houran et al., 2019a,b). Accordingly, there seems to be a core “encounter” phenomenon that can be described as a syndrome (Laythe et al., 2021a).

These episodes are not uncommon in the general population and have strong supernatural connotations for many people (Hill et al., 2018, 2019; Houran et al., 2020). Research indicates that singular or sporadic haunt-type experiences can be induced for clinical, leisure, research, or transpersonal purposes by means of *suggestion*–*expectancy effects* (Houran et al., 2020), *transcerebral magnetic stimulation* (Persinger et al., 2000), *creative dissociation* (Maraldi and Krippner, 2013), *psychedelic use* (Davis et al., 2020), *channeling activities* (Pederzoli et al., 2022), *ritual settings* (Caputo et al., 2021), and *environmental psychology* (Dagnall et al., 2020). However, individuals with recurrent encounter experiences or ghostly episodes over time and under naturalistic and spontaneous conditions possibly represent a more complex or nuanced process. We speculate that such instances involve the hypothesized concept of “Haunted People Syndrome” (HP-S) (for overviews, see O’Keeffe et al., 2019; Lange et al., 2020; Laythe et al., 2021a).

Following systems (or biopsychosocial) theory, Laythe et al. (2021a) used their grounded theory interpretation of recent empirical research to describe HP-S as an cognitive–affective phenomenon involving transliminal perceptions (“the right people”) that are structured due to attentional and perceptual mechanisms, and facilitated by transliminality-conducive environments (“the right settings”), which often produce a self-reinforcing loop (“psychological contagion”) that is contextualized and reinforced by attributions of external agency (“belief in the paranormal”) as a coping mechanism. Put succinctly, the interaction among sensory-somatic sensitivities, situational context, and social milieu prompts certain individuals to endorse paranormal agents or entities as the preferred explanation for the perceived complexity (i.e., ambiguities or anomalies) in their environments. This model essentially equates spontaneous ghostly episodes to some of the fundamental mechanisms that stoke outbreaks of mass (contagious) psychogenic illness (Lange and Houran, 1998, 1999, 2001a), although the flurries of symptom perception in HP-S appear to be mostly self -induced and -sustained.

Active haunt–type occurrences that are available for scientific scrutiny are quite rare, especially those involving dramatic somatic phenomena (see e.g., Nisbet, 1979; Amorim, 1990; Mulacz, 1999; Houran, 2002; Houran et al., 2019b,2002b; Taff, 2010; Ritson, 2020). But an account fortuitously came to our attention that allowed us to empirically test the practical utility and predictive validity of the current HP-S model with quasi-longitudinal data. This study thus compares the onset (*macro-phenomenology*) and contents (*micro-phenomenology*) of a particularly intense spontaneous case, which has persisted for over a decade, to the five proposed features (or recognition patterns: Carleton and Webb, 2012) of HP-S as outlined by Laythe et al. (2021a, 2022). We specifically hypothesized that the phenomenology of this ghostly episode—labeled the “San Antonio Disturbances”—would show that:

a. Transliminality and reinforcing Paranormal Beliefs mediate percipients’ anomalous experiences.
b. “Dis-ease” (notable but sub-clinical levels of stress) is a catalyst for the onset of anomalous experiences.
c. Diverse anomalous experiences are involved that exhibit temporal patterns suggestive of psychological contagion.
d. Attributions for the anomalous experiences align to the percipients’ biopsychosocial milieu.
e. Percipients’ anxiety levels relate to the nature, proximity, and spontaneity of their anomalous experiences.

## Synopsis of the San Antonio Disturbances

Correspondence with the afflicted family during introductions and early data collection quickly revealed that this case centered on the life story—or what could be deemed a narrative identity (Dunlop, 2017), personal myth (Krupelnytska, 2020), or illness narrative (Shapiro, 2011)—of the primary percipient named *Nell* (pseudonym, age 57). Secondary percipients included Nell’s daughter from her first marriage (*Jill*, age 37) and her second husband (*Rod*, age 70). The San Antonio family highlighted the anomalous events that have haunted them for the last ten years, but the case actually began in Nell’s childhood. Below we detail her psychosocial history from previous medical records and many days of structured interviews and impromptu discussions.

A biomedical (or generally skeptical) perspective might assume that Nell’s anomalous experiences are delusions or hallucinations from an unmanaged mental health issue (e.g., a thought or personality disorder) or medical condition (e.g., an acquired brain injury from trauma or stroke). However, the available evidence does not immediately support these speculations. Table 1 summarizes the independent findings from a Texas Board certified psychiatrist, who conducted a diagnostic impression of Nell on 20 March 2020. This type of assessment involves an interpretive statement based on previous and current evaluative data, which may or may not reference criteria in the *Diagnostic and Statistical Manual of Mental Disorders* (*DSM-5)* or *International Classification of Diseases* (*ICD-10*). The psychiatrist’s evaluation included letters from the family’s church, interviews with both Rod and Jill, as well as email communications and face–to–face assessment.

**TABLE 1:** Diagnostic impression of the primary percipient per independent assessment (dated 20 March 2020).

Diagnostic impression	Diagnosis given as Delusional Disorder (F 297.1), persecutory type, with bizarre (implausible content) continuous. Does not meet criteria for: chronic psychosis, schizophrenia, psychotic disorders, psychotic related mood disorders, substance induced psychoses, delirium, organic cases of acute paranoia, major minor neurocognitive disorders, malingering, factitious disorder, and personality disorders

**Contextual observations/etiological opinions**	Opines phenomena as likely hypnopompic and hypnogogic hallucinations, citing relationship between times of rest and phenomena as a potential explanation. States, “the subjective belief that they were experienced [the phenomena] as she recounts them is itself delusional”. Opines with regards to family experience of phenomena as a case of shared delusional disorder. Cites cognitive memory error as explanation for early childhood paranormal experiences of daughter and mother and misinterpretation of experiences. Notes no pharmacologic treatment. Anti-psychotics not recommended due to lack of cognitive symptoms, or psychotic symptomology beyond reported paranormal experience

**Recommendations**	Recommends counseling, with subject open to possibility of hypnopompic/hypnogogic experience, and culturally sanctioned supernational beliefs with confabulatory memories, and a debriefing. Notes belief system is entrenched, but overall functioning is deemed “good”

Nell was subsequently given the provisional diagnosis of delusional disorder, i.e., an exclusively present belief system that is not culturally congruent with medical science, while lacking all other symptoms of psychosis or mood related disorders. Personality and somatic disorders were further ruled out by the psychiatrist. Also relevant in the clinical report is the assumption of a shared delusional disorder that attributes the family’s anomalous or altered experiences to confabulatory memory or hypnogogic–hypnopompic episodes (cf. Hufford, 2001). We note that the psychiatrist’s explanation for Nell’s claims is culturally dependent on the assumption that her perceptions are incompatible (or impossible) within a medical model.

### Nell’s Early History of Encounter Experiences

Nell grew up in a family that spoke openly about paranormal experiences. In fact, her childhood home was allegedly haunted along with the two adjacent houses. She later explained how UFOs would also be frequently seen in the neighborhood, with many residents gathering together to watch them at night. However, the number of anomalous events that Nell reported during her Childhood and Teenage years was low. Table 2 presents an aggregated history of Nell’s encounter experiences starting in childhood, with one exceptionally memorable event involving her toy doll that reportedly “came to life and snarled at her.”

**TABLE 2:** Mean characteristics of the primary percipient’s history of encounter experiences.

	Number of memorable events	Event type^a^	Setting^b^	Context^c^ (% of experiences coinciding with distress or eustress)	Proximity^d^	Fear/anxiety^e^
Childhood	5	1.6	1.2	0	1.2	3
Teenage	11	1.5	1	0	1	3
Young adult	24	1.2	1.4	0.38 (Distress = 08% Eustress = 29%)	1.4	2
Adult—“Residence A”	13	1.2	1.4	0.92 (Distress = 43% Eustress = 43%)	1.3	2.2
Adult—post “Residence A”	315	1.8	1.7	0.37 (Distress = 27% Eustress = 10%)	1.5	1.9

*^a^Event Type: “subjective/psychological = 1, objective/physical = 2”.*

*^b^Setting: “experienced alone = 1, others present = 2”.*

*^c^Context: “notable stressors, emotions, or situations happening at the time = 1, no notable stressors, emotions, or situations happening at the time = 0”.*

*^d^Proximity: “event occurred inside personal space = 1, event occurred outside personal space = 2”.*

*^e^Fear/Anxiety: “not at all anxious/scared = 0, a little anxious/scared = 1, somewhat anxious/scared = 2, very anxious/scared = 3”.*

Although Nell never claimed to have imaginary companions (IC), her doll and some other toys appeared to be “personified objects.” Social scientists subsume this behavior under the IC rubric (Moriguchi and Todo, 2018), and both personified objects and traditional ICs have been linked to ghostly episodes or encounter experiences in childhood (Young, 2018; Laythe et al., 2021b; Little et al., 2021). Nell continued to have multiple encounter experiences throughout her teenage years and early adulthood. Overall, these early encounters tended to occur when she was alone. As examples, she talked about how “something” would sit on the bed towards her legs and stroke them, whereas many other times she would hear a male voice call out her name in a thick whisper from the stereo speakers.

### Nell’s Later History of Encounter Experiences

Table 2 shows that Nell’s anomalous experiences increased in frequency during Young Adulthood and then grew exponentially in later Adulthood. The central events in this case began in 2011 at “Residence A.” This was the first of the family’s five residences (“A to E”) due to successive moves in an attempt to escape the presumed paranormal activity. Nell explained the beginning of the family’s past 10-year saga this way:

One day we decided to look on the property that was directly in back of my house [Residence A]. We had a tall wooden privacy fence that enclosed my entire yard, sides, and back with a gate to the back far right. We [“Nell, Rod, Jill and Jill’s then boyfriend Warren”] went through that gate and there were hanging tree branches, tall weeds, and grass. It smelled like fresh horse manure about 20–30 feet into this area, but no horses were anywhere. We walked around and saw excess amounts of old leftover building supplies, bits, and pieces. I told the others that I was going home as I did not like it there, but they stayed to prowl around. A week or so later a man came to my front door and introduced himself. He was dressed in a suit-style black leather jacket, a black ribbed turtleneck shirt, gold chain and medallion, black pants, and boots—in scorching hot August weather in Texas! He spoke to my husband and had a very heavy Russian or Eastern European accent. I did not speak to him but stood in the background to listen. Rod said that this man told our neighbors and the mailman to keep off his property to avoid injuries or accidents. He left me with an “unnerving feeling.” Another time this same man also spoke to my daughter in her backyard saying it was good that she had guard dogs for protection. Soon after, the series of mysterious events began with a bite to my left breast while I was sleeping with my cat, and Rod was away working offshore on an oil rig. The next morning, I could see teeth marks above and below the areola. Jill saw it too, but we did not think to take a picture. The following night there was a loud crash at about 3 a.m. in the morning. It sounded like my huge China cabinet had been tipped over, a loud crashing sound that scared me something terrible. I got up to see what happened and saw the light in my office was on. This room is directly across from my bedroom, and it was filled with dense, deep amber-color whirling fog from floor to ceiling. I phoned Jill who lived next door to get over there and help me to get the cat and myself out. She came and also saw the fog. We booked it to her house, and I called Rod offshore to tell him to get home. He arrived about a day-and-a-half later, and the fog was still there and only in that room. The fog started to subside on the third day. Also, Jill had two German rottweiler blockhead dogs, and the male dog died of an apparent drug overdose afterwards. He was found in her backyard with blood coming out of its mouth. It all seemed like such strange timing. Nothing unusual was happening in our lives before and during this. Rod and I were working, and Jill and the kids seemed happy in their home … we all had dinners at my house and BBQs, nothing that stood out as anything abnormal. It was after the fog appeared that all hell broke loose. It seems to me that it came from that property, that something was released, being the only logical cause to me. I know it sounds odd, but this whole thing is very odd and to continue after all these years and seems to follow us and intensify more as time passes … and even when it seems to simmer down a bit, it will rear its ugly head and something new or different will start up. After so long, certain things you have to learn to accept and attempt to continue with life, but when it affects you physically as well that is a different situation. It causes you to dramatically age physically, mentally, emotionally and spiritually. Well, at least to me it has, and it is unfortunately very visible (J. Houran, personal communication, 14 June 2021).

As documented in more detail in a later section (cf. Table 6), subsequent anomalies experienced by Nell and her family included near daily occurrences of apparitions, sensed presences, negative feelings, threatening tactile sensations, unusual odors, and object displacements. They also claimed that their security camera recordings would often show “phantom voices and figures.” The family members reportedly experienced these and other anomalies both when they were alone and together. A marked upsurge in Nell’s encounters is attributable to the period “Post-Residence A.” This might suggest the presence of strong context effects, such as attentional or confirmation biases (Lange and Houran, 2001a). Experiences in Post-Residence A largely involved *O* phenomena that contrasted with her Childhood and Teenage accounts which were a mixture of *S/O* anomalies, and her Young Adult-Residence A experiences of mainly *S* events. Nell’s Childhood and Teenage experiences happened alone, her Young Adult and Adult-Residence A occurred both alone and with others, and the Post-Residence A experiences mainly occurred in the presence of others. Regarding proximity, most of Nell’s experiences during her Childhood and Teenage years reportedly happened within her personal space, whereas her Young Adulthood and Adulthood (Residence A and Post-Residence A) experiences occurred variously within and outside her personal space. Finally, Nell’s anxiety–fear was reportedly highest (“very anxious/scary”) during her Childhood and Teenage years, while the levels during her Young and Later Adulthood time periods were associated only “somewhat” with anxiety–fear.

## Method

### Case Information

On 31 May 2021, the first author was contacted by the Director of the “Paranormal Phenomena Research and Investigation” team after searching for names of “clinical parapsychologists” on the Parapsychological Association website. This group was working with “a female from Texas who was looking for assistance with a haunting situation” and they concluded from their initial evaluation that the case required a researcher with counseling expertise because it involved “a small amount of anomalous activity” combined with pronounced “psycho-social factors” (personal communication to J. Houran). The first author then enlisted the assistance of the second author, who is a forensic psychologist and Director of the Institute for the Study of Religious and Anomalous Experience (ISRAE)—a registered not-for-profit dedicated to academic research and public education.

The authors accepted the referral after first determining that the circumstances likely did not involve a mental disorder with religious themes, as well as gaining approval from the afflicted family for the arrangement. The outline and goals for this study were subsequently approved by Ethics Committee at ISRAE to guarantee compliance with ethical guidelines proposed for this subject area (Baker and O’Keeffe, 2007), including informed consent in writing by each family member pertinent to data collection and its subsequent use for research and reporting purposes (Gavey and Braun, 1997). The family’s participation was entirely voluntary, involved no financial compensation, and could be stopped at any point. We evaluated and synthesized material from three sources: (a) records provided by the three family members who constituted the primary and secondary percipients in this case, (b) copies of findings and conclusions from a prior investigation by the independent group noted above, and (c) clinical, historical, psychometric, and environmental data that we collected first-hand as outlined below.

### Procedure

Laythe et al.’s (2021a, 2022) five presumed features of HP-S were clearly specified prior to the data collection and analysis. Similar to pre-registered studies, this tactic aimed to control for undisclosed flexibility that can lead to revisionist or false discoveries (Nosek et al., 2018). Qualitative studies are popular in the psychological literature on anomalous experiences (e.g., Childs and Murray, 2010; Drinkwater et al., 2013; Eaton, 2019), but some researchers may dismiss such findings as anecdotal information in the absence of rigorous scientific controls and numerical data. In contrast, quantitative research minimizes subjectivity in favor of objectivity by deductively forming a hypothesis derived from theory. Controlled, objective testing and experimentation ultimately supports or rejects the hypotheses under consideration. For these reasons, we adopted a mixed methods approach that primarily used quantitative analyses supplemented with qualitative insights as appropriate.

We further structured our study using the five-step Evidence-Based Practice (EBP) framework (Sackett et al., 1996; Guyatt et al., 2000; Straus et al., 2011). This involves (a) converting information needed into answerable clinical questions; (b) tracking down best evidence for answering the questions; (c) critically appraising the evidence for validity, impact, and applicability; (d) integrating the evidence into clinical decision-making; and (e) assessing the prior steps to improve future efforts. Accordingly, we administered planned and unplanned measures at different points of our study depending on the theoretical or clinical direction the case took. The tasks or assessments that we used were intentionally divvied over time so not to overwhelm the afflicted family. Overall, we spent approximately six months working with the family in research and therapeutic contexts.

After making the initial introductions and obtaining informed consent, we asked the three family members to complete psychometric measures #1–6 described below prior to the authors’ in-person visits to the family’s residence. As explained in a later section, we administered assessment #7 *post-hoc* to clarify pertinent background information in this case. All measures were administered online. Next, we separately asked Nell to prepare a chronological log of her most memorable encounter experiences from childhood to present-day. This might seem an infeasible or incredulous task, but she was quite confident in her memory of these past and ongoing events. The instructions for this exercise requested that she include the following six details for each of the major anomalous experiences that she could readily recall: (a) *Time Period* (“Childhood, Teenager, Young Adult, Adult-Residence A, Adult- Post-Residence A”), (b) *Anomalous Event* (selected from items 1-32 on the Survey of Strange Events), (c) *Setting* (anomalous event experienced “alone or with others”), (d) *Context* (“notable negative emotions, stress, or dis-ease at the time of the anomalous experience; notable positive emotions, stress, or dis-ease at the time of the anomalous experience; or no notable emotions, stress, or di-ease at the time of the anomalous experience”), (e) *Proximity* (anomalous events occurred “inside or outside her personal space”), and (f) *Anxiety/Fear* level perceived during each anomalous event (i.e., “very anxious/scared, somewhat anxious/scared, a little anxious/scared, or not at all anxious/scared”). This “event diary” exercise was likewise completed online. Finally, we conducted two separate and extended visits with the family over the course of three, non-consecutive days. These particular interactions aimed to (a) cross-check or clarify their previous responses to the questionnaires, (b) observe their family dynamics, (c) personally assess audio and video “evidence” of the haunting activity that the family had assembled over recent months, and (d) collect environmental readings of physical variables that might contribute to some or all of the family’s anomalous experiences (cf. Dagnall et al., 2020; Jawer et al., 2020).

### Measures

(1) The 16-item, Rasch scaled version (Lange et al., 2000a) of the *Revised Paranormal Belief Scale* (RPBS) remedies Tobacyk’s (1988, 2004) original 26-item, Likert-based form (seven response categories anchored by “strongly disagree to strongly agree”), with an artificial structure of seven factors due to differential item functioning, i.e., sex and age response biases. Correcting these measurement problems with a “top-down purification” procedure using Modern Test Theory, Lange et al. (2000a) showed that the RPBS comprises only two, moderately correlated belief subscales that seemingly reflect different issues of control.

Specifically, New Age Philosophy (NAP) (11 items, Rasch reliability = 0.90) seems related to a greater sense of control over interpersonal and external events (e.g., “Some individuals are able to levitate (lift) objects through mental forces”), whereas Traditional Paranormal Beliefs (TPB) (5 items, Rasch reliability = 0.74) seem more culturally-transmitted and beneficial in maintaining social control via a belief in magic, determinism, and a mechanistic view of the world (e.g., “Through the use of formulas and incantations, it is possible to cast spells on persons“). Several studies support the construct validities of these two subscales (Houran et al., 2000, 2001; Houran and Lange, 2001c), which both have a *mean* of 25 (*SD* = 5).

(2) *Revised Transliminality Scale* (RTS: Lange et al., 2000b; cf. Houran et al., 2003b) is is a Rasch version of Thalbourne’s (1998) original 29-item, true/false scale (Form B). Twelve items from the original scale are excluded from the scoring due to age and gender biases. However, the remaining seventeen test items constitute a unidimensional Rasch (1960/1980) scale with a Rasch reliability of 0.82. These 17-test items, which share a common underlying dimension, span seven domains: Hyperesthesia, (fleeting) Hypomanic or Manic Experience, Fantasy-Proneness, Absorption, Positive (and perhaps obsessional) Attitude Towards Dream Interpretation, Mystical Experience and Magical Thinking. RTS scores (*M* = 25, *SD* = 5) consistently predict different syncretic cognitions, somatization and hypochondriacal tendencies, and lower psychophysiological thresholds (Houran et al., 2002a; for overviews, see Evans et al. (2019), Lange et al. (2019)).

3. *Survey of Strange Events* (SSE: Houran et al., 2019b). This is a 32-item (T/F), Rasch-scaled measure of the overall intensity of a ghostly account or narrative via a checklist of subjective and objective (*S/O*) events or experiences inherent to these anomalous episodes [e.g., sample items include “I felt odd sensations in my body, such as dizziness, tingling, electrical shock, or nausea (sick in my stomach” and “I saw objects breaking (or discovered them broken), like shattered or cracked glass, mirrors or housewares,” respectively]. Specifically, the SSE’s Rasch hierarchy represents the probabilistic ordering of *S/O* anomalies according to their endorsement rates but rescaled into a metric called “logits.” Higher logit values signify higher positions (or progressively lower likelihood of endorsement) on the Rasch scale (Bond and Fox, 2015). We refer readers to our previous papers for details on the development and utilization of this instrument (Houran et al., 2019a,b, 2021).

Rasch scaled scores range from 22.3 (=raw score of 0) to 90.9 (=raw score of 32), with a mean of 50, *SD* = 10, and a Rasch reliability = 0.87. Higher scores correspond to a greater number and intensity of anomalies that define a percipient’s experience. Supporting the SSE’s content and predictive validities, Houran et al. (2019b) further found that the phenomenology of “spontaneous” accounts (i.e., ostensibly “sincere and unprimed”) differed significantly from “control” narratives from “primed conditions, fantasy scenarios, or deliberate fabrication.” Follow-up studies with the SSE also support its value for thematic analyses of qualitative reports (O’Keeffe et al., 2019; Lange et al., 2020; Laythe et al., 2021b; Little et al., 2021).

4. *Desirability for Control Scale* (DCS: Burger and Cooper, 1979) has 20 items that measure individual differences in the general level of motivation to control the events in one’s life. The desire for control is a general personality trait, relevant to many behaviors studied by both social and clinical psychologists. Much research and theory suggests that an increase in perceived control is preferred and results in positive reactions, whereas a decrease in control is not desired and leads to negative reactions. Sample items include “I try to avoid situations where someone else tells me what to do” and “I wish I could push many of life’s daily decisions off on someone else.” The scale is reported to have good internal consistency (0.80), test-retest reliability (0.75), and discriminant validity from measures of locus of control and social desirability.

5. *I-E Scale* (Rotter, 1966) measures generalized internal–locus of control, or the extent to which individuals believe that they can control events that affect them. Externals believe that outcomes are beyond their control, whereas internals believe they can influence outcomes. Researchers have both modified the scale in various ways over the years (Marsh and Richards, 1986) and debated its dimensionality (Marsh and Richards, 1987), but we opted for the original 29-item (unidimensional), forced-choice version (with six filler items) that is the most often used (Wang and Lv, 2017). Sample items include “Many of the unhappy things in people’s lives are partly due to bad luck” and “When I make plans, I am almost certain that I can make them work.” Scores range from “0” (internality) to “23” (externality), and the measure shows satisfactory psychometric properties from a Classical Test Theory perspective (e.g., Rotter, 1975).

6. *Highly Sensitive Person Scale* (HSPS: Aron and Aron, 1997) assesses physiological reactivity to stimuli in the environment (e.g., “Are you easily overwhelmed by strong sensory input?”) and subtle reactivity (e.g., “Do you become unpleasantly aroused when a lot is going on around you?”). Respondents respond to 27 items, indicating how much the situation described in each applies to them, using a 7-point Likert scale ranging from “1 (not at all) to 7 (extremely).” Scores are normally calculated as the average of the 27 ratings, but alternatively the scale’s total value can be used to create a dichotomous variable representing two groups (low vs high sensitivity). Note that the total value has also been used as a continuous variable in some research (e.g., Jagiellowicz et al., 2011). Several studies support the tool’s reliability and content validity (e.g., Aron and Aron, 1997; Smolewska et al., 2006).

Respondents answer a series of questions, indicating how much the situation described in each applies to them, using a 7-point Likert scale ranging from “1 (not at all) to 7 (extremely).” Example questions are: “Are you easily affected by other people’s moods?”, “Do you find loud noises uncomfortable?”, and “Are you aware of subtle changes in your surroundings?” The scale gives a total value, which is used to create a dichotomous variable representing two groups (low vs high sensitivity), but the total value has also been used as a continuous variable in some research (e.g., Jagiellowicz et al., 2011). Several studies support the tool’s reliability and content validity (Aron and Aron, 1997; Smolewska et al., 2006).

(7) *Survey of Traumatic Childhood Events* (STCE: Council and Edwards, 1987) is a 30-item retrospective measure of the occurrence and frequency of 11 types of aversive childhood experiences, i.e., intrafamilial sexual abuse, extrafamilial sexual abuse, intrafamilial physical abuse, loss related to a friend, loss related to the family, isolation, personal illness or accident, parental divorce/separation and abortion/miscarriage, (extrafamilial) assault, loss of the home, and robbery. Responses on the STCE are made on a five-point scale (1 = “none” to 5 = “more than ten”). Note that items #29 and 30 are multiplied together to give a single variable; item 29 is a trauma occurrence variable, whereas item 30 gives the length of time this trauma lasted. Also, some items are potentially sensitive, such as those concerning sexual abuse, so the instruction sheet was designed with particular sensitivity in mind to such ethical issues.

There are no published psychometric data on the STCE—only descriptive information (e.g., Irwin, 1992; Thalbourne et al., 2003; Dorahy et al., 2004)—and research on the prevalence and adulthood sequelae of childhood trauma has been criticized for the use of assessment instruments with unknown psychometric properties (Scher et al., 2001). Nevertheless, the STCE seemed the best instrument for our purpose as it covers perhaps the broadest range of traumatic events of any of the available childhood trauma questionnaires.

## Results

The following subsections compare relevant details of this case to the five presumed recognition patterns of HP-S. This format should help readers to better follow our arguments and make clearer distinctions in the array of technical or nuanced information considered here. Some analyses are statistically underpowered, so we encourage readers to mainly focus on the direction and size of the effects from attenuated *r* statistics. Further, degrees of freedom are clearly marked in all analysis in order to guide the reader with regards to statistics that are suggestive but lacking in robust sample sizes.

### Preliminaries

Haunt-type occurrences always involve the risk of fraud for various motivations (Roll, 1977; Nickell, 2001), but we neither have evidence of deliberate deceit nor do we suspect a factitious component here. Indeed, an evaluation of key patterns in Nell’s anomalous experiences using the Decision-Tree Process in Houran et al. (2019b, p. 180) indicated that this case can be classified with 90% accuracy as a “non-illicit episode.” This means that the events addressed in this paper are likely not to be explicitly fraudulent. However, this heuristic does not clarify whether the case is genuinely spontaneous versus rooted in active priming or pure imagination.

As the family became increasingly comfortable during our interactions, they started to elaborate on their psychosocial and medical histories, anomalous experiences, and the quality of their familial relationships. These spontaneous and sporadic disclosures indicated that Nell almost certainly minimized or omitted some of the information on the questionnaires in an attempt at impression management. This is a response bias that reflects the tendency for individuals to answer questions in a manner that will be viewed favorably by others, such as over-reporting “good or desirable” behavior or under-reporting “bad or undesirable” behavior. Thus, it poses serious problems when conducting research with self-reported information that pertains to unusual, atypical, or “unlikely” experiences, or in response to demand characteristics (Merckelbach et al., 2017). Specifically, it appeared that Nell wanted to emphasize the intense and mysterious nature of her experiences while not coming across as “crazy” (for discussions of this issue, see Roxburgh and Evenden, 2016a,b). Our initial findings and interpretations therefore often required re-examination and synthesis beyond the data originally collected with the questionnaires.

### Feature 1: Transliminal Perceptions Reinforced by Paranormal Belief

Transliminality is currently described as “a hypersensitivity to psychological material originating in (a) the unconscious, and/or (b) the external environment” (Thalbourne and Maltby, 2008, p. 1618). This perceptual-personality variable thus parallels and correlates with Hartmann’s (1991) mental boundary construct (Houran et al., 2003a), as well as involves Aron and Aron’s (1997) concept of sensory processing sensitivity. Note that Transliminality ostensibly acts as both a state and trait variable (Evans et al., 2019), meaning its effects can fluctuate with variations in an individual’s situational context. High transliminals show lower psychophysiological thresholds or neurological gating across various settings, but those with low-to-average levels are expected to show an increased *frequency* or *intensity* of such perceptions primarily under conditions of strong sensory or emotional stimulation (Lange et al., 2000b,^2019^; Evans et al., 2019). In practice, the preceding patterns suggest that high transliminals tend to facilitate or generate their altered or anomalous experiences, whereas low-to-average transliminals often require external catalysts for such effects.

Nell scored one standard deviation above the mean on the RTS, whereas Rod and Jill had slightly above-average scores (see Table 3). Consistent with the transliminal model (Laythe et al., 2018; Ventola et al., 2019), the family’s RTS scores showed a moderate correlation [*r*(13) = 0.36, *p* = 0.17] with their respective SSE scores across Residences A–E. This suggests that the family’s anomalous experiences are, in part, linked to high trait levels of Transliminality. Additionally, we discuss below how ongoing disruptions in the family’s biopsychosocial environment likely bolstered their thin boundary functioning. Thus, both state and trait Transliminality are potential factors in this case.

**TABLE 3:** Perceptual-personality profiles of the afflicted family members.

	Revised Transliminality Scale (*M* = 25, *SD* = 5)	Highly Sensitive Person Scale (*M* = 4.09, *SD* = 0.83)	Rasch—Tobacyk’s Revised Paranormal Belief Scale^a^ (*M* = 25, *SD* = 5)	Desirability for Control Scale (*M* = 100, *SD* = 10)	Rotter’s Locus of Control Scale (*M* = 11.5, *SD* =)
**Nell** (Primary experient)	30.9	*M* = 2.85 (Raw total = 77)	*NAP* = 31.89 *TPB* = 43.24	87	6
**Rod** (Secondary experient)	25.7	*M* = 4.63 (Raw total = 125)	*NAP* = 28.24 *TPB* = 39.23	95	10
**Jill** (Secondary experient)	25.7	*M* = 3.22 (Raw total = 87)	*NAP* = 31.89 *TPB* = 29.02	87	6

*^a^NAP, New Age Philosophy; TPB, Traditional Paranormal Beliefs.*

Moreover, replicating prior research (Laythe et al., 2018, 2021a), Table 3 clearly implicates Paranormal Belief in the family’s anomalous experiences. Notably, each family member scored above-average on both the TPB and NAP varieties of PB, although TPB showed generally stronger levels and Nell was highest on both belief types. This implies that the entire family had strong foundational levels of Paranormal Belief that included both “internalized and externalized” supernatural forces but with an emphasis on external or autonomous agents. However, the family members’ respective SSE scores across Residences A–E, correlated *r*(13) = 0.47, *p* = 0.07 with NAP and *r*(13) = 0.02 (*p* = 0.94) with TPB. This skew towards NAP is due to the daughter’s patterns; the role of TPB becomes evident [*r*(8) = 0.69, *p* = 0.03] when scores for Nell and Rod are considered by themselves as the sole occupants of Residences A–E.

### Feature 2: Dis-ease as a Catalyst for Anomalous Experiences

“Dis-ease” refers to a non-pathological alteration in waking experience, i.e., an individual’s state of “ease” becomes notably imbalanced or disrupted. Studies suggest that anomalous experiences attributed to ghosts or poltergeists are often idioms of distress or broader dis-ease (e.g., Rogo, 1982; Houran et al., 2002a; Ventola et al., 2019). This pattern likewise applies to religious stigmata phenomena (Kechichian et al., 2018), and we should similarly note that Drinkwater and colleagues have found that percipients’ interpretations of paranormal experiences are significantly mediated by their perceived anxiety (Drinkwater et al., 2013, 2017). However, dis-ease does not always entail “distress” (or negative emotions or stressors, e.g., abuse or injury, death of a family member, or financial problems) but also can mean “eustress” (or positive emotions or stressors, e.g., marriage, starting a new job, or buying a new home). Some stressors can be positive or negative depending on a host of factors, e.g., holiday seasons or the birth of a child. We refer readers to Ventola et al. (2019, pp. 146–157) for a discussion of these nuances in ghostly episodes.

Using Nell’s retrospective log, which included all of her individual accounts of experiences across the five residences (*n* = 267 entries), there was a negative correlation [*r*(265) = −0.25, *p* < 0.001] between the number of Nell’s anomalous experiences across her life periods and the general presence of eustress or distress, perhaps demonstrating a normalization of the phenomena by Nell over time (but see below). Yet, the presence of dis-ease did have a small and negative association [*r*(265) = -0.22, *p* < 0.001) with Nell’s categories of *S/O* experience throughout her life, i.e., distress or eustress tended to coincide with subjective rather than objective phenomena. These findings largely undermine the idea of dis-ease as either a necessary or consistent catalyst in this case. However, a number of other patterns suggest that this variable had indeed played an active and important role here.

To begin, recall that the patterns in Table 2 imply that dis-ease coincided with some of Nell’s anomalous experiences as a Young Adult (around a third of the time) and then again as an Adult primarily at Residence A (where some source of stress was reportedly a prominent factor), and to lesser extent across Residences B–E. In these select instances ostensibly linked to dis-ease, the Young Adult period mostly referenced eustress, Residence A showed a balance of eustress and distress, whereas Residences B–E tended towards distress. That is, for some reason, Nell’s anomalous experiences have become increasingly connected with the presence of distress.

Next, it is difficult to rectify some of Nell’s questionnaire responses against her subsequent verbal reports. For example, Nell claimed to have “no notable stress, emotions, or situations” during Childhood (cf. Table 2), even though during later interviews she detailed a string of impressionable, if not influential, events that she experienced in relatively rapid succession at three years old. In fact, Nell reportedly remembers these quite vividly, i.e., (a) she witnessed along with her mother and grandparents a gruesome accidental death in May 1968 that was documented in the local newspaper, (b) the following June her grandfather died of natural causes, and (c) in July of the same year she turned four years old (i.e., a self-reported example of potential eustress). We suspected that events like this produced sustained dis-ease in Nell; an idea we sought to verify with the STCE measure (see section “Method,” #7).

However, her STCE score of “8” was unremarkable and overall indicated a relatively low level of childhood trauma that in more extreme forms otherwise predict dissociation-schizotypy related phenomena (Irwin, 1992, 1996; Lawrence et al., 1995; Giesbrecht et al., 2007; Gibson et al., 2019). Of course, Nell’s score might also reflect impression management or even repression of unreported events. Dis-ease was reportedly associated with anomalous experiences during Young Adult (around a third of the time) and Residence A, where notable dis-ease was allegedly a prominent factor. Consistent with this interpretation, there was a small but positive correlation [*r*(265) = 0.11, *p* = 0.40] between the presence of dis-ease and the intensity of Nell’s haunt-type perceptions (i.e., SSE item logits) across her life periods.

Finally, Nell’s struggle with ongoing bouts of dis-ease featured prominently in her personal myth or illness narrative. During our visits and other interactions, she often talked about her life being constantly filled with distress from personal disappointments or medical challenges. This was such a frequent theme in her conversations with us that we asked her to list the most noteworthy examples. Clinical details are omitted here in the interest of confidentiality, but Table 4 suggests that the most frequent dis-ease events (as judged most memorable by Nell) occurred during the periods of Childhood and Adult Post-Residence A. In about 17% of Nell’s listed events, the dis-ease referenced perceptions that we know she deemed as “ghostly”. But most often her distress represented biopsychosocial variables that apparently lacked a paranormal context (83% of the listed events). Taken altogether, the role of dis-ease in this case is reasonably confirmed albeit the actual prevalence or strength of its influence is unresolved.

**TABLE 4:** Primary percipient’s most noteworthy dis-ease events across life periods.

Childhood period
Nine-months old—Rubella (German Measles)
Cat scratch fever five times
Witnessed accidental death of an unknown woman
Death of her grandfather
Stepped on a rusty nail that went through foot, tetanus shot
Car accident car hit turning into driveway flew and hit head on driver window age 10
Fell out of tree house backwards age 10, nothing but breathless
Hit between eyes pitching at softball game
Racing mini-bike down alley and friend ran into me, thrown into fence
Hand slammed in car door
Right calf burned from brothers side pipes on car (left clovers pattern burn)
Age 12–24-year old brother slammed my face into car windshield
**Teenage period**
Age 13 serious elbow damaged from flying rock
Speed skating and caught right knee on brick entrance to the floor, knee puffed up
First panic attack at 14, in Minnesota at oldest brother’s house, he slapped me in the face to try to make it stop
**Young adult period**
Age 20 got locked in elevator expecting Jill
Tumor size of grapefruit to right ovary, pain like knife to upper thigh, difficulty walking, miracle it disappeared. with ob gyn confirming most likely my daughters twin that never developed causing significant pain to thigh when trying to walk
Age 22 fell down a flight of stairs with heels on and fractured both feet
Age 25 came up from sitting and caught top of forehead on cabinet causing deep dent and cut
Age 32 fractured right foot again at robs mom house on front steps
Age 33 got shingles, but never had chicken pox
**Adult-residence A period**
Top of left hand cut at jenika’s house from thing holding her arm throwing holy water that turned to blood
Started to get psoriasis on inner right ankle one area repeat from the stress
Car accident and hit head on left-side against window and hurt right shoulder, bruised
Hurt lower back again carrying jenika from car to house after knee surgery she had
**Adult post-residence A period**
Fractured right foot while trying to pack house on phoenix street
Aneurism in arm
Boxes falling on me for no reason, skin tears, bruises
Walking and hit by car in parking lot
Lacunar stroke from nine-day migraine
Busted interior of right knee on door jamb chasing my daughter, swollen up to size of small watermelon, took four months to recover
First time pneumonia from casino in eagle pass
Top of right hand split open
Hand slammed in door ×2
2nd degree burn to left breast from pot in sink that splashed by itself
Woke up to a bite mark to inner left thigh
Scratches to upper back
Awoke to find red ligature mark three-quarters around my neck
Chair fell backwards and landed exactly on left big toe at quick and nail, pain and dent and bruising, nail almost grown out, june of last year
Packing boxes in garage and things started falling on me. bruises cuts, knots
Again boxes falling and cut from boxes on arms and hands
Trouble coping with her daughter’s sexual identity issues
Kicked out my daughter and grandchildren out of my house due to moral argument

### Feature 3: Diverse *S/O* Anomalies With Temporal Patterns Suggesting Psychological Contagion

The HP-S model implies that encounter-prone individuals perceive a spectrum of *S/O* symptoms over time as opposed to having isolated occurrences or a limited range of perceptions (Houran et al., 2019b). This contradicts the idea that percipients merely perceive (or have experienced) a single anomaly, such as “sensing a presence,” “hearing a physical knocking,” or “seeing a ghost.” Moreover, the HP-S concept contends that the detection of anomalous (or ambiguous) stimuli spreads within and across individuals similar to a contagious illness due to expectancy effects or Baader-Meinhof illusions. That is, transliminal perceptions can promote perceptual or confirmation biases as percipients search for additional evidence of their attributions, interpretations, or general beliefs. Several studies have correspondingly found temporal patterns in symptom perception that implicate psychological contagion or memetic-type processes in ghostly episodes (e.g., Houran and Lange, 1996; Lange and Houran, 2001a,b; Laythe et al., 2017; Drinkwater et al., 2019; Langston and Hubbard, 2019; Tashjian et al., 2022).

Consistent with expectations, this case contained a diverse set of *S/O* anomalies that were perceived over time. Table 5 shows that 11 (or 69%) out of the family’s set of 16 collective experiences across Residences A–E (including Residence F for daughter Jill) showed above-average “haunt intensity” (or perceptual depth) per Houran et al.’s (2019b) norms. Nell especially noted a wide array of encounter experiences throughout her life, starting with a haunted childhood home in which she had a frightening IC-type experience. Another striking event that occurred in her Adulthood (i.e., Spring of 2021) strongly paralleled reports of “alien abductions” (see e.g., Mack, 1994). Particularly, Nell went to bed fairly early (still daylight), dozed off, and suddenly felt as if she awoke to a dark room with soft lights in the walls. She was lying on a very cold, silver-colored metal table, with a bright white light shining on her. Nell was reportedly immobile apart from being able to move her eyes. Overhead she then saw a pencil-thin light about six inches from her face. The table would slide back-and-forth several times under this light while she laid there. She neither saw, heard, nor smelled anything or anyone, and the duration of the experience was unknown.

**TABLE 5:** Family members’ SSE scores (“Haunt Intensity”) across the successive residences.

	SSE: Residence A	SSE: Residence B	SSE: Residence C	SSE: Residence D	SSE: Residence E	SSE: Residence F
**“Nell”/Mother** (Primary experient)	55.3	60.7	51.0	59.6	60.7	*n/a*
**“Rod”/Husband** (Secondary experient)	54.3	48.6	49.8	49.8	54.3	*n/a*
**“Jill”/Daughter** (Secondary experient)	61.9*	57.5*	58.5*	49.8*	45.9*	59.6

*Visitations to these residences only.

**TABLE 6:** Endorsement of SSE items between residences and witnesses.

		*Symptom and rarity spontaneous cond.*			*Correspondence between houses*						*Between witnesses*			
		*COMMON*	*LOGIT*	*P*	*Res. 1*	*Res. 2*	*Res. 3*	*Res. 4*	*Res. 5*	*Ave.*	*Mother*	*Dad*	*Daught.*	*Ave.*
SSE15	1	Deja Vu	-1.65	**0.84**	0.33	0.33	0.00	0.00	0.33	**0.20**	0.60	0.00	0.00	**0.20**
SSE14	2	Sensed Presence	-1.59	**0.83**	0.33	0.00	0.00	0.33	0.00	**0.13**	0.20	0.00	0.17	**0.12**
SSE17	3	Unrecognizable Sound	-1.17	**0.76**	*1.00*	*1.00*	*1.00*	*1.00*	*1.00*	**1.00**	*1.00*	*1.00*	*1.00*	**1.00**
SSE20	4	Cold Area	-0.80	**0.69**	0.33	0.00	0.33	0.33	0.67	**0.33**	*0.20*	*0.80*	*0.17*	**0.39**
SSE29	5	Breeze	-0.73	**0.67**	*1.00*	*1.00*	*1.00*	*0.67*	*0.67*	**0.87**	*1.00*	*1.00*	*0.50*	**0.83**
SSE16	6	Recognizable Sound	-0.62	**0.65**	*0.67*	*0.67*	*0.67*	*0.33*	*0.33*	**0.53**	1.00	0.00	0.67	**0.56**
SSE25	7	Erratic Electronics	-0.62	**0.65**	*0.33*	*0.33*	*0.33*	*0.33*	*0.67*	**0.40**	1.00	0.20	0.00	**0.40**
SSE1	8	Non Descript Visual Form	-0.62	**0.65**	*1.00*	*1.00*	*1.00*	*1.00*	*1.00*	**1.00**	*1.00*	*1.00*	*1.00*	**1.00**
SSE7	9	Negative Feeling	-0.60	**0.65**	*0.67*	*0.67*	*0.33*	*1.00*	*0.67*	**0.67**	*0.80*	*0.80*	*0.33*	**0.64**
SSE31	10	Non Hostile Touch	-0.55	**0.63**	0.67	0.33	0.00	0.00	0.33	**0.27**	*0.60*	*0.00*	*0.33*	**0.31**
		***LESS COMMON***												
SSE3	11	Obvious Apparition	-0.51	**0.62**	*0.67*	*0.67*	*0.33*	*0.67*	*0.33*	**0.53**	*0.60*	*0.40*	*0.67*	**0.56**
SSE2	12	Alive Looking Apparition	-0.47	**0.62**	*0.67*	*0.33*	*0.67*	*0.33*	*0.67*	**0.53**	*0.60*	*0.40*	*0.67*	**0.56**
SSE8	13	Odd Body Sensations	-0.47	**0.62**	*0.33*	*0.67*	*0.33*	*0.33*	*0.33*	**0.40**	0.60	0.60	0.00	**0.40**
SSE22	14	Object Teleport	-0.10	**0.52**	0.00	0.00	0.33	0.00	0.00	**0.07**	0.00	0.00	0.17	**0.06**
SSE23	15	Object Movement	-0.05	**0.51**	0.00	0.33	0.00	0.00	0.00	**0.07**	0.20	0.00	0.00	**0.07**
SSE26	16	Recording of Image	-0.05	**0.51**	0.00	0.33	0.33	0.00	0.00	**0.13**	0.20	0.00	0.33	**0.18**
SSE13	17	Communication with X	0.03	**0.49**	0.33	0.00	0.00	0.00	0.00	**0.07**	0.00	0.20	0.00	**0.07**
SSE4	18	Pleasant Odor	0.04	**0.49**	*0.67*	*0.67*	*0.33*	*0.33*	*0.67*	**0.53**	0.60	0.00	1.00	**0.53**
SSE6	19	Positive Feeling	0.10	**0.48**	*1.00*	*0.67*	*0.67*	*0.33*	*0.67*	**0.67**	*1.00*	*0.40*	*0.67*	**0.69**
SSE19	20	Rec. of Unrecognizable Sound	0.16	**0.46**	*1.00*	*1.00*	*1.00*	*1.00*	*0.67*	**0.93**	*1.00*	*1.00*	*0.83*	**0.94**
SSE18	21	Rec. of Recognizable Sound	0.24	**0.44**	0.33	0.33	0.33	0.00	0.00	**0.20**	0.00	0.00	0.67	**0.22**
SSE5	22	Unpleasant Odor	0.42	**0.40**	*0.67*	*0.67*	*0.33*	*0.67*	*0.33*	**0.53**	0.00	0.60	1.00	**0.53**
SSE32	23	Threatening Touch	0.44	**0.39**	*0.67*	*0.67*	*0.67*	*0.33*	*0.33*	**0.53**	1.00	0.00	0.67	**0.56**
		***RARE***												
SSE28	24	Object Breakage	0.51	**0.38**	*1.00*	*1.00*	*1.00*	*1.00*	*1.00*	**1.00**	*1.00*	*1.00*	*1.00*	**1.00**
SSE24	25	Object Levitation	0.65	**0.34**	*0.67*	*0.33*	*0.33*	*1.00*	*0.67*	**0.60**	*0.20*	*0.80*	*0.83*	**0.61**
SSE21	26	Hot area	0.72	**0.33**	*0.33*	*0.67*	*0.33*	*0.33*	*0.33*	**0.40**	0.60	0.00	0.67	**0.42**
SSE10	27	Possession	0.84	**0.30**	*1.00*	*1.00*	*0.67*	*0.67*	*0.67*	**0.80**	*1.00*	*0.60*	*0.67*	**0.76**
SSE27	28	Plumbing Malfunctions	0.90	**0.29**	0.00	0.00	0.00	0.00	0.00	**0.00**	0.00	0.00	0.17	**0.06**
SSE11	29	Mythical Type Beings	1.07	**0.26**	*0.67*	*0.67*	*0.67*	*0.67*	*1.00*	**0.73**	*1.00*	*0.20*	*1.00*	**0.73**
SSE9	30	Taste	1.08	**0.25**	0.33	0.00	0.00	0.00	0.00	**0.07**	0.00	0.00	0.17	**0.06**
SSE12	31	Folklore Type Beings	1.61	**0.17**	0.33	0.00	0.00	0.33	0.00	**0.13**	0.20	0.20	0.00	**0.13**
SSE30	32	Fires	1.71	**0.15**	0.00	0.00	0.00	0.00	0.33	**0.07**	0.00	0.20	0.17	**0.12**

*Bolded figures indicate raw probabilities of occurrence, and underlined figures denote high rates of phenomena consistent across residences.*

From 2011 to 2021 alone, Nell has perceived sensed presences, non-descript visual forms, alive-looking apparitions, mystical-type beings, and folklore-type beings. There was also an event at Residence A that was reminiscent of a MIB encounter, as well as aspects of newly recognized types of encounter experiences like “group- (or gang) stalking” (Lange et al., 2019; O’Keeffe et al., 2019). These patterns challenge the idea that encounter experiences constitute separate phenomena with different sources or mechanisms (e.g., Gauld and Cornell, 1979; Solfvin and Williams, 2021). Moreover, themes of negativity, threat, and persecution have dominated Nell’s *S/O* experiences to the extent that she has often doubted her own sanity akin to self–imposed gaslighting (for discussions of related themes in encounter experiences, see Drinkwater et al., 2019; O’Keeffe et al., 2019; Lange et al., 2020).

Regarding the other aspect of Feature 3; however, a lack of data suitable for time series analyses made it infeasible to directly assess contagion or memetic processes affecting the family (see Houran and Lange, 1996; Lange and Houran, 2001a,b; Drinkwater et al., 2019). We can nonetheless still test some broad patterns that might indirectly suggest the influence of psychological contagion, namely whether the family has (i) SSE scores that increase sequentially across Residences A–E, (ii) positive correlations among their SSE scores (i.e., similar *perceptual depth* of haunt experiences), (iii) similar strength and direction of *successive variations* in their SSE scores across Residences A–E, and (iv) positive correlations among the SSE items that they specifically endorsed (i.e., *perceptual congruence* in their experiences).

The HP-S model recognizes psychological contagion as a measurable concept that presumably involves the instigation of successive (episodic) experiences due to expectancy effects on individuals or across a group of people. But we must underscore that in many cases, such as mass psychogenic illness (Sapkota, 2017), the exact mechanisms of contagion are not fully understood. As our study seeks to map the reported signs and symptoms in haunt-type episodes, it is notable that contagion (as a sharing or commonality of experiences or symptoms) is present not only across multiple residences, but between percipients. However, it is currently unclear the extent to which “congruence of experiences” derives purely from the influence of cuing or priming.

Analysis of Table 5’s underlying data found that the idea of contagion had mixed support per hypothesized patterns (i)–(iv) noted above. Pattern (i) was generally confirmed, although pattern (ii) instead showed opposite effects, i.e., Nell had a near zero correlation with Rod [*r*(3) = 0.02] but a moderately strong inverse correlation with Jill [*r*(3) = −63, *p* = 0.26], Rod and Jill likewise showed a small negative correlation [*r*(3) = −0.15, *p* = 0.81]. Thus, the family tended to show *near*-*zero* to *opposite* congruence in their respective haunt intensities across the successive residences. But Nell and Jill’s experiences certainly had a conspicuous connection that remains to be clarified. Next, pattern (iii) likewise tended to show opposite effects to expectations. Fluctuations in Nell’s SSE scores across the five residences had a low to moderate negative correlation with Rod’s SSE fluctuations [*r*(2) = −0.36, *p* = 0.63] and an even stronger negative correlation with Jill’s SSE fluctuations [*r*(2) = −0.96, *p* = 0.04]. However, the variability of Rod and Jill’s SSE scores across the successive residences were positively correlated [*r*(2) = 0.17, *p* = 0.82].

While SSE scores varied across the percipients, Table 6 supports hypothesized pattern (iv) by clearly highlighting 12 separate SSE items that were both experienced by all percipients and across all five residences (See Table 6, italics). These items included four commonly-witnessed phenomena (i.e., “cold area, breeze, non-descript visual forms, and negative feelings”), four less common items (i.e., “obvious apparition, alive looking apparition, positive feelings, and recordings of unrecognizable sounds”), and four rarely-endorsed items (i.e., “object breakage, object levitation, partial or full possession, and seeing mythical-type beings”). Thus, approximately 37.5% of potential SSE items were consistently perceived both across multiple locations and between all experients in this case.

The putative contagion pattern that emerges from Tables 5, 6 is complex. On the one hand, Nell showed a general increase in the number of encounter experiences over her life, and she and Rod both had broad but slight increases in perceived haunt intensities across the sequential residences. These trends are arguably consistent with contagion at the experient level. However, the family had negative to near-zero correlations among the perceptual *depth* and *contents* of their experiences across the sequential residences, and the daughter’s SSE scores generally decreased over time. However, other qualitative information suggests that contagion or memetic effects were involved but primarily confined to individual family members.

Similar to studies showing cuing–priming effects in paranormal contexts (Houran and Lange, 1996; Laythe et al., 2017; Houran et al., 2020), Nell and Jill both independently reported a flurry of *S/O* experiences during the two-week time period preceding our in-person visits. Specifically, Nell claimed that their computers would “act up” when they attempted to email us information. She remarked that, “Believe it or not this stuff happens when trying to send photos or videos pertaining to this nightmare.” Nell would also occasionally report that “things are increasing,” “new types of things are happening,” and “this thing is really putting up a fight.” The daughter similarly reported increased sleep disturbances, sensed presences, and inexplicable sounds at her own home (Residence F).

Despite a lack of temporal sync between the occurrence of events, Nell and Jill’s inverse relationship suggests that one or the other, at a specific time, was more likely to report anomalous experiences. Table 6 further implies that specific *S/O* perceptions were contagious, albeit not necessarily at the same time for each percipient. It is evident that, if we examine these anomalous experiences solely from a contagion perspective, reporting of one particular feature of the SSE by a family member was followed by others also reporting it. The combined findings from Tables 5, 6 arguably suggest the presence of contagion–memetic processes in this case, although the timing of these perceptions seemed to vary as a function of the interpersonal relationships between Nell, Rod, and Jill.

### Feature 4: Attributions for *S/O* Anomalies Align to Percipients’ Biopsychosocial Environment

Quali-quantitative observations affirmed this recognition pattern. Table 2 showed that PB was a strong and ready variable to contextualize the family’s apparent transliminal experiences. Particularly, both Nell and Rod showed above-average levels of PB, although they scored consistently higher on TPB than NAP. It would be expected therefore that they would interpret their anomalous experiences in terms of *external* agency, such as dogmatic, religious-oriented concepts or forces. This was confirmed during our in-person visits whereby each room in Nell’s house was found to be heavily decorated with different forms of Christian iconography. She also reported experiencing “positive” meaningful coincidences (e.g., repeatedly seeing the number “22” in everyday situations) that she interpreted as the presence or influence of “angels.” This religiosity carried over from her childhood and young adulthood, where she felt like a “martyr” due to the many sacrifices or burdens she endured for others. In short, Nell construed the prevalence of dis-ease in her life as directly linked to the conviction of her religious faith.

It thus made sense that Nell and Rod attributed the *S/O* phenomena to a “a malicious entity that is trying to terrorize her [Nell] because she is so religious.” In fact, Nell specifically identified the persecutory spirit as “Beelzebub”—a name used by some Abrahamic religions for a major demon or even Satan. More to this point, Nell stated that one tactic that would often would temporarily halt her anomalous experiences would be to play the “Pie Jesu Domine” dona eis (est) requiem by vocalist Charlotte Church. Later in the paper we discuss at length more evidence supporting Feature 4, as Nell’s strong TPB hindered our intervention strategy for the family (see “Clinical Complications During the Investigation”). Conversely, Jill’s higher NAP versus TPB score suggests a stronger belief or influence of *internal* agency, such as one’s own “psychic” ability. This agreed with Jill’s self-description as “an extremely protective mother of her children in the face of the paranormal activity.” She accordingly considered herself strong or empowered enough to manage whatever was causing her anomalous experiences.

### Feature 5: Percipients’ Anxiety Levels Are Related to the Nature, Proximity, and Spontaneity of *S/O* Anomalies

Threat (and agency) detection (Freeman et al., 2002; Gaynor et al., 2013; Brett et al., 2014; Jelić and Fich, 2018; Coelho et al., 2021; Tashjian et al., 2022)—or Hypersensitive Agency Detection Device (HADD, see e.g., Barrett, 2000, 2004; Atran, 2002; Guthrie, 2013)—likely influences HP-S in several ways that we have discussed previously (Drinkwater et al., 2021; Laythe et al., 2021a). First, anomalies might be judged as more or less frightening depending on their degree of *spontaneity*. Increasingly anxious or fearful reactions are likely when anomalous perceptions occur unexpectedly. An accompanying decline in overall mental health might also occur with individuals who have a strong “need for control” (Langer and Rodin, 1976; Leotti et al., 2010). That said, other studies suggest that a low “desirability for control” is associated with poorer reactions (Burger and Cooper, 1979).

Next, there is the degree to which percipients interpret specific *S/O* anomalies as inherently threatening due to their *nature*, e.g., the more physical the events, the more dangerous they might seem. Finally, we expect that the more *proximal* the anomalies are to one’s personal space, the more intense or prevalent the corresponding interpretations of anxiety, fear, threat, or persecution. Personal space is the region surrounding individuals that they regard as their psychological territory and physical domain. Most people value their personal space and feel discomfort, anger, or anxiety when this space is encroached (Welsch et al., 2019). Thin boundary functioning is further expected to facilitate threat (agency) detection in that Transliminality arguably supports predictive coding (Evans et al., 2019), which Anderson (2019) has argued can effectively account for HADD-related behavior.

Given her strong level of TPB, Nell profiled unsurprisingly as having a low desirability for control (cf. Table 3). These trends clearly align to a personal ideology that emphasizes an *external* locus of control regarding life events. The anxiety–fear levels coinciding with Nell’s anomalous experiences were only somewhat acerbated by ongoing or precursor stress as suggested by a very low but significant Spearman correlation [*rho*(265) = 0.10 *p* = 0.03] between Nell’s anxiety–fear levels and concurrent dis-ease in her life. Further, her anxiety-fear levels showed a similar relationship [*rho*(265) = 0.16, *p* = 0.003] in accordance with the intensity of specific *S/O* anomalies (measured by their Rasch logit values, cf. Houran et al., 2019b, p. 173). In other words, the more “intense” the anomalous events were in a psychometric sense, the more anxiety-fear Nell tended to experience. Table 7 further shows that anxiety–fear levels have small but consistent relationships with anomalies that occurred (a) inside her personal space, (b) when she was alone, and (c) involved *subjective* (psychological) experiences versus *objective* (physical) events (cf. Table 2).

**TABLE 7:** Primary percipient’s anxiety–fear levels during anomalous experiences correlated to situational factors.

Contextual factors	Anxiety-fear level (Spearman *rho*)
**Dis-ease** (distress or eustress)	0.10
**Proximity** (1 = inside, 2 = outside personal space)	–0.15
**Setting** (alone = 1, others = 2)	–0.26
***S/O* event type** (*S* = 1, *O* = 2)	–0.26
***S/O* logit value** (Rasch scaled intensities of specific events: Houran et al., 2019b)	0.16

## Clinical Complications During the Investigation

Case studies of presumed HP-S ideally include interventions to help individuals understand and cope with their anomalous experiences, starting with the educational task of normalizing these occurrences for percipients. Laythe et al. (2021a) further discussed a range of approaches to ameliorate the (a) frequency or intensity of experients’ symptom perception, and/or (b) anxieties related to the anomalous or threatening nature of the *S/O* phenomena. Other authorities have also offered useful guidance to clinical practitioners (Hastings, 1983; Targ and Hastings, 1987; Coly and McMahon, 1993; Chadwick et al., 1996; Brett et al., 2007; Murray, 2012; Alton, 2020; Webb, 2021). We emphasize that social desirability biases can be major confounds when assessing and addressing potential HP-S. Based on our dealings with Nell’s family in this respect, we recommend that researchers or clinicians not administer screening inventories or psychological assessments prior to establishing strong rapport with the percipients to safeguard against impression management (see Roxburgh and Evenden, 2016a,b; Drinkwater et al., 2019). Further, questionnaires that address controversial beliefs or experiences might be better administered via empathetic, in-person interviews rather than standard administrative methods that could cause individuals to feel judged on their mental acuity.

Nell also responded enthusiastically to the attention shown to her during our investigation. This spotlight might have met psychological needs that were otherwise unsatisfied within her family dynamic. But she also wanted from us an outright validation of her interpretation for the anomalous events, which prompted her to resist our explanations and related options for relief. Specifically, we assigned Jill and Nell visualization exercises that emphasized “protection” in combination with “mindfulness” meditations. These exercises were rooted within a religious ideological framework to which the family could relate, in the hopes of reducing emotional stimulation that fueled the family dynamic and ostensibly fostered both transliminality and Nell’s histrionic or catastrophizing reactions to the anomalous experiences generated by the transliminality. Note that this approach paralleled Jalal’s (2016) use of focused-attention meditation combined with muscle relaxation therapy to relieve fits of sleep paralysis, which is an experience of immobility that often includes terrifying hypnogogic or hypnopompic hallucinations with paranormal undertones (cf. Hufford, 2001).

The tactic reportedly provided appreciable relief in the short-term, but the family regarded the exercises as too tedious to sustain. We next recommended that Nell explore “Eye Movement Desensitization Reprocessing” (EMDR; for an overview, see Castelnuovo et al., 2019). This evidence-based psychotherapy draws on the Adaptive Information Processing model that posits much of psychopathology is due to the maladaptive encoding or incomplete processing of traumatic or disturbing adverse life experiences (Hase et al., 2017). EMDR has shown corresponding efficacy for psychiatric and somatic disorders with comorbid psychological trauma (Valiente-Gómez et al., 2017), and, thus, it might also be effective for aspects of HP-S.

We think that our recommendations eventually failed for two reasons. On one hand, Nell sought a quick remedy to their situation. On the other hand, and consistent with gaslighting effects in haunt accounts (Drinkwater et al., 2019), Nell strongly resisted any interpretation that differed with her belief that evil spirits were the primary source of the *S/O* anomalies. In fact, Nell’s reactions to our conclusions in this case strongly paralleled the behaviors of naïve research subjects who observe staged “paranormal” demonstrations. For instance, participants sometimes remember witnessing manifestations (even physical events like object displacements) that actually never happened (Wiseman et al., 2003). Moreover, proponents of psychic phenomena tend to rate such staged demonstrations as more paranormal than disbelievers, and these beliefs often persist even *after* debriefing (French, 1992; Hergovich, 2004; Smith, 1992/1993; Wiseman and Morris, 1995). Apparently for some people, the paranormal is the preferred explanation even when such beliefs conflict with the available evidence (for a discussion, see Houran and Lange, 2004).

Nell was careful not to completely dismiss our conclusions and recommendations, but she quickly pushed for a consultation with a spiritual medium to validate her stance on the anomalous experiences. This shift appeared to us as a form of “doctor shopping (or hopping),” which involves patients who seek multiple clinicians or second opinions (Sansone and Sansone, 2012; Velma et al., 2014; Lane, 2020) often as a way “to interpret, regulate, and mediate various forms of self-understanding and activity” (Brinkmann, 2017, p. 170). This behavior can be particularly aggravated when an individual is dealing with medically unexplained symptoms (de Zwaan and Müller, 2006). She eventually contacted a local psychic and Reiki practitioner, who concluded that her family was probably cursed in some way. Nell understandably seized on this agreeable opinion as it fit with her TPBs and explicitly confirmed her conviction that an external, malevolent agent was responsible for the family’s haunting.

Research shows that metaphysical—or spiritistic—oriented interventions sometimes alleviate haunt-type experiences (Roll, 1977; Lucchetti et al., 2011; Storm and Tilley, 2020). Rather than proving the reality of the paranormal, of course, successful outcomes in this respect can be explained as psychodramas, demand characteristics, or placebo effects (for discussions, see Storm and Tilley, 2021; Laythe et al., 2022). Only time will tell whether a “psychic intercession” benefits Nell’s family. However, our prognosis is not optimistic. Two previous house blessings by Catholic priests reportedly failed to stop the *S/O* anomalies. This seemingly contradicts her high TPB, but expectancy effects from these rituals were perhaps nullified by strong criticisms and resentments towards the Catholic church that Nell voiced to us. Symptom relief appears further unlikely without a stabilized family dynamic, especially as related to Nell’s attention-seeking behavior that might hint at a broader martyr or victim complex—or perhaps even covert narcissism (i.e., narcissistic personality disorder)—meant to elicit sympathy, love, admiration, loyalty, or even guilt from her family and broader social support network. We do not assert here that mental illness explains this case; only that our observations lead us to suspect that some type of condition or temperament issue has moderated her reactions to the anomalous experiences. For more information on clinical theory and practice in this context, we refer readers to Rabeyron’s (2022) detailed overview, discussion, and recommendations.

## Discussion

Key aspects of the San Antonio Disturbances generally fit the five proposed recognition patterns of HP-S. That is, quali-quantitative analyses affirmed several predictions from Laythe et al. (2021a, 2022) about the features and dynamics of ghostly episodes which manifest spontaneously and recurrently to certain people. The strongest alignment to the HP-S model was the associations between the family’s anomalous experiences and their elevated levels of Transliminality (*sensitivity*) and Paranormal Belief (*ideology*). This agrees with the interactionist view that bridges the Experiential Source versus Cultural Learning views of anomalous experience (for discussions, see Laythe et al., 2018; Lange et al., 2019). Indeed, growing evidence suggests that ghostly episodes like the present case involve mutually-reinforcing contributions from both unusual perceptions and the cognitive frameworks that percipients use for meaning-making (Houran et al., 2002b; Wiseman et al., 2002; French et al., 2009; Langston and Hubbard, 2019).

The anxiety or fear reported by the primary percipient showed patterns that broadly align to principles of threat (and agency) detection. But this does not mean there is nothing to learn in this area and as applied specifically to religious- or supernatural- oriented contexts. For instance, recent work (Tashjian et al., 2022) demonstrates the relevance of (a) social dynamics (friends vs. strangers) for *tonic arousal* (i.e., intrinsic arousal that fluctuates on the order of minutes to hours.) and (b) subjective fear and threat predictability for *phasic arousal* (i.e., a respondent state of vigilance increment of short endurance and dependent upon the stimulus conditions of novelty and others). People’s demographic characteristics can further influence their fears of particular supernatural topics (Silva and Woody, 2022).

A related issue concerns the main sources of fear and anxiety with *S/O* anomalies. For instance, Naij and van Elk (2017) talked about the difference between “prior expectations” formed by interaction with the environment (e.g., instruction, cultural transmission, learning, and reliance on source credibility) and “evolved priors” that were presumably selected by a process of natural selection. We should emphasize that fear is not the only possible response to ghostly episodes. Often, percipients also reference a sense of “enchantment” that disrupts normal waking experience with a sudden, unexpected, or profound awareness that ultimately culminates in a transformative feeling of connection to a “transcendent agency or ultimate reality” (Holloway, 2010; Drinkwater et al., 2022; Houran et al., 2022). The interplay among all these dynamics should be explored in-depth, as they may mediate contagion effects.

Now the roles of dis-ease and psychological contagion as the instigators or facilitators of the anomalous experiences in the present case received mixed support. These possible inconsistencies might derive from imprecise or incomplete data or insufficient methodologies applied to such data. Accordingly, future research should explore several alternative explanations. Particularly, it might be that the presumed features of HP-S (a) are neither simultaneously involved, nor all required in the process; (b) do not necessarily constitute the same process in every case; or (c) are not completely defined in their components or mechanisms. This latter issue might particularly pertain to psychological contagion, given that we observed decent-sized effects but typically skewing opposite to predictions. This could suggest that the underlying mechanisms and subsequent effects of psychological contagion, or cuing in general, are more complex or nuanced than currently understood.

Nevertheless, we can characterize Nell’s encounter experiences as: (a) mostly prevalent in adulthood, (b) manifesting both inside and outside her personal space, (c) involving a mixture of *S/O* anomalies, with a recent flood of *O* events from the use of audio-video technology she has used to document perceived anomalies, (d) inducing moderate levels of anxiety or fear, (e) often occurring in the presence of others with similar belief structures, and (f) ensuing within a context of strong and distressing family dynamics that have been normalized and unaddressed in a clinical sense. Considering all the information and evidence available to us, we conclude that this case represents the symptoms and manifestations of thin (permeable) mental boundary functioning in the face of unfavorable circumstances or overstimulating environments and subsequently acerbated by poor emotion regulation, histrionic or catastrophizing reactions, and active confirmation biases.

This study has several important limitations. First, our inferences and conclusions were based on limited data that likely produced statistically non-significant outcomes in some instances. Therefore, replications are critically needed to affirm our findings and their implications. This includes the use of large datasets and corrections for multiple observations as opposed to our more liberal case study approach. Second, we cannot cross–check the veracity of the information in this case. Confounds can arise with naturally “noisy” data, including latency effects with retrospective accounts, as well as omissions, embellishments, or fabrications of some or all the salient details. Indeed, impression management, at least on Nell’s part, was undoubtedly a constraining factor at times. Despite these potential shortcomings, we contend that our findings cannot be fairly dismissed as artifacts of overly cursory or exploratory analyses or overreaching interpretations. Indeed, hypothesis-testing with quantitative methods identified patterns that often were consistent with theory-driven predictions.

Moreover, these same outcomes are probably not as small or subtle as the statistics might suggest. Readers should be mindful that attenuated coefficients are artificially weakened by the unreliability inherent to all psychometric measures. Therefore, the true effect sizes related to the five recognition patterns found here are likely to be much larger than they appear (for discussions, see e.g., Jensen, 1998; Lange et al., 2019). That said, we were not experimentally blind to the hypotheses when collecting and interpreting the data in this case. As a result, we could be criticized for not fully controlling for experimenter biases that possibly influenced our approaches or conclusions (Holman et al., 2015). Pre-registered studies by independent researchers guided by our framework and methodologies should help to address this concern.

Third, several other pertinent psychometric measures could have been administered to further refine our understanding of witness psychology in paranormal contexts, such as *ambiguity tolerance* (Lange and Houran, 2001a), *aberrant salience* (Irwin et al., 2014), *idiopathic environmental intolerance* (Witthöft et al., 2008), *schizotypal tendencies* (Cicero et al., 2021), and particularly in this case, the variables of *allostatic load* (i.e., the cumulative burden of chronic stress and life events, Guidi et al., 2021) and *negative urgency* (i.e., the tendency to act rashly when distressed, Settles et al., 2012; see also Joyner et al., 2021). Percipients’ receptivity to *psychological contagion* might also be explored deeper with measures such as the Gudjonsson (1984) Suggestibility Scale or the Absorption Scale to gauge a person’s tendency to become immersed within sensory experiences (Tellegen and Atkinson, 1974). Beyond modeling the predictors or mediators of HP-S related perceptions and reactions, this line of research might be particularly useful for identifying effective treatment options for experients. Here is where qualitative methods might serve as a valuable augment to gain richer knowledge about how percipients construct meaning from their experiences and likewise how experiences affect individuals (cf. “HP-S Feature 4: Attributions for *S/O* Anomalies Align to Percipients’ Biopsychosocial Environment”).

Lastly, our study considered only the psychosocial aspects of the family’s anomalous experiences versus their potentially parapsychological nature. Some researchers reject this latter line of inquiry (e.g., Nickell, 2001; Reber and Alcock, 2020), while others embrace it (e.g., Roll, 2003; Maher, 2015). Blanket dismissals of this controversial viewpoint in terms of fraud, hype, noise, or confusion are arguably simplistic, misguided, and counterproductive to ongoing model–building and theory–formation in consciousness studies. Indeed, we would be remiss not to mention that transliminality positively correlates with several indicators of putative psi (Thalbourne and Houran, 2003; Thalbourne and Storm, 2012; Ventola et al., 2019). Furthermore, we obtained some unusual environmental readings during the present investigation that will be explored in separate research (cf. Laythe and Houran, 2018; Dagnall et al., 2020; Laythe et al., 2021c). And finally, different processes likely underlie the occurrence of anomalous experiences versus the attributions used to describe or explain them (Ross et al., 2017). Thus, poorer cognitive functioning or mental wellness can sometimes be a reaction or consequence of having anomalous or altered experiences versus the precursor or cause (Inglis and Storm, 2021).

That said, the HP-S model neither requires nor negates the ontological reality of parapsychological mechanisms. Our collective research instead suggests—irrespective of potential psi—that spontaneous ghostly episodes like the San Antonio Disturbances are a pronounced psychological phenomenon at the crossroads of belief- and boundary- structures and reflective of dis-ease states or circumstances (Laythe et al., 2021a, 2022). We thus encourage scientists across all disciplines to take haunt and poltergeist reports seriously and to explore their “blue ocean” of data using fresh and impartial perspectives. Studying these percipients and their biopsychosocial environments should help to clarify processes for coping and meaning-making relative to the complex issue of spirituality in mental health (O’Reilly, 2004; Johnson and Friedman, 2008; Koenig, 2012) and the associated continuum in the general population along which normal and extraordinary forms of perception and cognition may be mapped (Persinger and Makarec, 1993; Claridge, 1997; Evans et al., 2019).

## Data Availability Statement

The non-confidential raw data and supplementary materials related to this study are on file at ISRAE and available to qualified researchers, https://www.israenet.org/. The other raw data supporting the conclusions of this article will be made available by the authors, without undue reservation.

## Ethics Statement

The studies involving human participants were reviewed and approved by Ethics Committee, Institute for the Study of Religious and Anomalous Experience. The patients/participants provided their written informed consent to participate in this study.

## Author Contributions

JH: conceptualization, project administration, investigation, formal analysis, and writing—original draft preparation, review, and editing. BL: conceptualization, investigation, formal analysis, and data curation. Both authors read and approved the final manuscript.

## Conflict of Interest

JH was employed by company Integrated Knowledge Systems. The remaining author declares that the research was conducted in the absence of any commercial or financial relationships that could be construed as a potential conflict of interest.

## Publisher’s Note

All claims expressed in this article are solely those of the authors and do not necessarily represent those of their affiliated organizations, or those of the publisher, the editors and the reviewers. Any product that may be evaluated in this article, or claim that may be made by its manufacturer, is not guaranteed or endorsed by the publisher.
